# The relations between constipation and characteristics of intestinal morphologies by colonoscopy

**DOI:** 10.3389/fgstr.2023.1255129

**Published:** 2023-09-11

**Authors:** Yuting Xu, Qili Xiao

**Affiliations:** Zhongshan Hospital of Traditional Chinese Medicine Affiliated to Guangzhou University of Traditional Chinese Medicine, Zhongshan, China

**Keywords:** colonoscopy, characteristics of intestinal morphologies, constipation, tortuousness, dissociation

## Abstract

**Background:**

Constipation is commonly diagnosed throughout the world, and it is typically associated with various factors. However, data on the characteristics of intestinal morphologies linked with constipation are scarce. We examined the association between the characteristics of different intestinal morphologies and constipation.

**Patients and methods:**

Between March 2020 and February 2021, we enrolled 510 patients from the Affiliated Zhongshan Hospital of Guangzhou University of Chinese Medicine into two groups: 260 in the constipation group and 250 in the control group. Of these patients, intestinal morphology characteristics obtained via colonoscopy were compared and analyzed.

**Results:**

There were meaningful differences between the cohorts based on the intestinal morphology characteristics of tortuousness (*p < *0.001) and dissociation (*p < *0.001). In addition, a significant difference in characteristics was determined for either both intestinal morphologies (*p < *0.001) or only tortuousness without any other conditions (*p*=0.015), but there was no significant difference between the two groups with respect to only dissociation without any other conditions (*p *= 0.077). A subgroup analysis was performed on statistically significant variables—gender (*p < *0.001), age (*p = *0.002), and operation time (*p < *0.001)—and the results showed that regardless of the subgroup analysis, there was a statistically significant difference in tortuousness between the two groups. In addition, there were significantly differences in dissociation between the groups for elderly men and those with a longer operation time.

**Conclusion:**

Compared with the general population, people with the intestinal morphologies of dissociation and, in particular, tortuousness seem to experience constipation more frequently.

## Introduction

Constipation is a common symptom across the world, affecting multiple aspects of a person’s health and their quality of life. The prevalence of constipation varies according to the diagnostic criteria used, but estimates put its prevalence between 2.6% and 26.9% (1). The Rome diagnostic criteria are the most commonly accepted standard, recognizing constipation as a subset of functional bowel disorders (2). The main clinical manifestations include difficulty in defecation, low defecation volume, decreased defecation frequency or incomplete defecation, and related discomfort. Constipation is a complicated condition and its pathogenesis is multifactorial (2, 3). There are various factors that are associated with constipation. The most frequently cited associated risk factors for constipation are female gender and advanced age (1, 3–5).

For patients with constipation as the only indication (i.e., without “high-risk symptoms” such as rectal bleeding, weight loss, and iron deficiency anemia), the guidelines do not explicitly recommend colonoscopy (6–8). In recent years, a considerable number of studies have addressed colonoscopy for patients with constipation as the sole clinical feature to investigate the association of constipation with colorectal cancer (CRC), colorectal polyps, and other significant pathological findings. A significant association between colorectal cancer and constipation has been observed (9–11), while other studies have shown the prevalence of neoplastic lesions to be low (12, 13). However, these recent studies were limited to colonoscopy diagnostic results, ignoring the analysis of relationships between other elements of the colonoscopy procedure and constipation. Data on the characteristics of intestinal morphologies associated with constipation are scarce.

The focus of this investigation was to summarize and analyze intestinal morphologies in constipation patients who underwent anesthetized colonoscopy. A subgroup analysis was conducted on common risk factors, such as female gender and advanced age, to provide further exploration for the study. The study posed the following question: “Do the characteristics of intestinal morphologies as determined by colonoscopy affect constipation or not?”

## Patients and methods

### Patients

#### Subject recruitment

From March 2020to February 2021, patients at the Affiliated Zhongshan Hospital of Guangzhou University of Chinese Medicine who underwent a painless colonoscopy were prospectively recruited and then divided into a constipation group and control group. The inclusion criteria were as follows:

1. Patients should have indications but no contraindications for painless colonoscopy.
2. Patients were of any gender and aged between 18–75 years.
3. Patients had signed the written consent form for examination and treatment.
4. Patients in the constipation group met the ROME IV diagnostic criteria for diagnosing constipation (14, 15). These are based on the characteristics of feces, personal bowel habits, and difficulty in defecation. A decrease in defecation frequency is the main criterion: constipation is generally defined as defecation every 2–3 days or less (or <3 times a week).
5. Patients in the control group had no gastrointestinal symptoms, including abdominal pain, abdominal distension, borborygmus, and abnormal defecation.

The following exclusion criteria were also applied:

1. Patients who were allergic to intravenous anesthesia or had serious adverse reactions.
2. Patients with a history of previous abdominal surgery.
3. Patients with psychosis, critical illness, cognitive impairment, or who were using hemp-based drugs.
4. Patients who underwent other invasive procedures prior to colonoscopy on the same day.
5. Patients who refused the scheduled treatments.

A total of 522 patients, constituting two cohorts, completed colonoscopy examinations. All patients experienced a painless colonoscopy administered by an experienced endoscopist who had performed 2,000–3,000 colonoscopies each year for at least 5 years. Of this group, 271 patients had been diagnosed with constipation based on the Rome IV criteria and 251 were healthy subjects with normal bowel habits (14, 15). Twelve patients were excluded from data analysis as they were diagnosed with CRC by colonoscopy. Consequently, we enrolled 510 patients in the study (260 in the constipation group and 250 in the control group).

In this prospective study, baseline and clinical data, comprising the patient’s name, gender, age, BMI, visit number, operation time, Boston bowel preparation score (BBPS), intestinal morphologies (such as tortuousness, long-windedness, dissociation, loops, peristaltic mucous membrane, and stenosis), terminal ileum intubation, and diagnosis [including enteritis (which showed in a colonoscopy as mucosal congestion and edema, without inflammatory bowel disease), colonic diverticulum, and intestinal polyps), were collected and recorded. All patients signed the written consent form for examination and treatment. Approval was obtained from the Institutional Review Board (the local Ethics Committee of the hospital) and the registration number for this study was 2019ZSZY-LLK-039.

### Definition of characteristics of intestinal morphologies by colonoscopy

The characteristics of different intestinal morphologies, as determined by colonoscopy, were defined as follows:

1. Tortuousness: no straight path can be observed in at least two intestinal segments.
2. Long-windedness: after completion of intubation of the colonoscopy to the ileocecal end/ileocecal part, the colonoscopy measures a length of at least 100 cm.
3. Dissociation: the front end of the colonoscope cannot be accurately inserted into the intestines, even if the colonoscope body is kept straight. That is, even when the mirror body is straightened, forcefully poking the mirror does not change the image.
4. Loops: the intestine twists around itself, and the colonoscope body hits the intestine in such a way that the colonoscope cannot pass through, while sharp curves can be observed (16).

### Statistical analyses

Data management and statistical analyses were performed using SPSS version 22.0 (SPSS, Chicago, IL, USA). Quantitative data with a normally distribution and homogeneity of variance are presented as the mean ± standard deviation, using the *t*-test for comparison. Non-normally distributed quantitative data are presented as the median and interquartile range (IQR), using the Wilcoxon rank-sum test. Differences in categorical data between groups were determined by the chi-square test or Fisher’s exact test. A *p*-value < 0.05 was considered to be significant.

## Results

Between March 2020 and February 2021, we enrolled 510 patients (260 in the constipation group and 250 in the control group) into the study. Comparisons of the variables in the propensity model between the two groups of patients are summarized in **Table 1**. There were statistically significant differences between the two groups in terms of gender (*p < *0.001), age (*p = *0.002), and operation time (*p < *0.001), but not for any other factors. In addition, the diagnosis of enteritis showed a tendency toward a statistically significant difference between groups, but did not reach significance (*p = *0.066).

**Table 1:** Comparison of clinical characteristics of 510 patients who underwent painless colonoscopy.

Variables	Overall population [n=510 (%)]	constipation group [n= 260(%)]	control group [n=250 (%)]	*x^2^*/*t*/*Z*	*P*
Gender				40.773	<0.001
Male	259(50.8)	96(36.9)	163(65.2)		
Female	251(49.2)	164(63.1)	87(34.8)		
Age [year, median (IQR)]	45.5(36.0∼54.0)	47.0(38.0∼58.0)	44.5(34.0∼51.3)	-3.037	0.002
BMI [kg/m^2^, median (IQR)]	23.2(21.1∼25.7)	22.9(20.8∼25.3)	23.6(21.3∼26.0)	1.660	0.097
BBPS [median (IQR)]	8.0(7.0∼8.0)	8.0(7.0∼8.0)	8.0(7.0∼8.0)	0.944	0.345
Operation time [min, median (IQR)]	12.0(10.0∼15.0)	13.0(10.0∼17.0)	12.0(10.0∼13.0)	-5.036	<0.001
Enteritis				3.371	0.066
Yes	397(77.8)	211(81.2)	186(74.4)		
No	113(22.2)	49(18.8)	64(25.6)		
Colonic diverticulum				0.117	0.732
Yes	22(4.3)	12(4.6)	10(4.0)		
No	488(95.7)	248(95.4)	240(96.0)		
Intestinal polyp				0.355	0.551
Yes	171(33.5)	84(32.3)	87(34.8)		
No	339(66.5)	176(67.7)	163(65.2)		
The cecal intubation				0.963	0.510
Yes	509(99.8)	259(99.6)	250(100.0)		
No	1(0.2)	1(0.4)	0(0)		

A comparison of the characteristics of intestinal morphologies determined by colonoscopy is shown in **Table 2**. There were meaningful differences between cohorts for the intestinal morphologies of tortuousness (*p < *0.001) and dissociation (*p < *0.001). Meanwhile, a significant difference between cohorts was observed for either a combination of tortuousness and dissociation (*p < *0.001) or tortuousness alone without other conditions (*p = *0.015) However, no significant difference between groups was observed for dissociation without any other conditions (*p = *0.007).

**Table 2:** Comparison of the characteristics of intestinal morphologies by colonoscopy definition.

Characteristics	Overall population [n=510 (%)]	constipation group [n= 260(%)]	control group [n=250 (%)]	*Z*	*P*
Tortuousness				-4.880	<0.001
Yes	77(15.1)	59(22.7)	18(7.2)		
No	433(84.9)	201(77.3)	232(92.8)		
Long-windedness				-1.354	0.176
Yes	18(3.5)	12(4.6)	6(2.4)		
No	492(96.5)	248(95.4)	244(97.6)		
Dissociation				-3.987	<0.001
Yes	150(29.4)	97(37.3)	53(21.2)		
No	360(70.6)	163(62.7)	197(78.8)		
Loops				-0.936	0.349
Yes	16(3.1)	10(3.8)	6(2.4)		
No	494(96.9)	250(96.2)	244(97.6)		
Both tortuousness and dissociation				-3.900	<0.001
Yes	32(6.3)	27(10.4)	5(2.0)		
No	478(93.7)	233(89.6)	245(98.0)		
Only tortuousness without other conditions				-2.435	0.015
Yes	37(7.3)	26(10.0)	11(4.4)		
No	473(92.7)	234(90.0)	239(95.6)		
Only dissociation without other conditions				-1.770	0.077
Yes	102(20.0)	60(23.1)	42(16.8)		
No	408(80.0)	200(76.9)	208(83.2)		

Referring to the World Health Organization (WHO) 2020 age classification standards, the participants were divided into three age groups: 18–44 years old (young), 45–59 years old (middle-aged), and over 60 years old (elderly). Furthermore, based on median operation time, the participants were divided into those with an operation time less than or equal to 12 minutes, and those with an operation time greater than 12 minutes. Subgroup analysis was conducted for statistically significant variables—gender, age, and operation time—and the results showed that regardless of the subgroup analysis, there was a statistically significant difference in tortuousness (all *p*-values < 0.05) between groups. In addition, there was a statically significant difference in dissociation for men (*p < *0.001), elderly people (*p < *0.001), and those with a longer operation time (*p = *0.001). The subgroup/sensitivity analysis for the characteristics of intestinal morphologies determined by colonoscopy is shown in **Table 3**.

**Table 3:** Subgroup/Sensitivity Analysis of the characteristics of intestinal morphologies by colonoscopy definition.

Variables	Tortuousness			Dissociation			Both tortuousness and dissociation			Only tortuousness without other conditions		
	constipation group	control group	*P*	constipation group	control group	*P*	constipation group	control group	*P*	constipation group	control group	*P*
Gender (%)												
Male	14(58.3)	10(41.7)	0.024	38(58.5)	27(41.5)	<0.001	8(80.0)	2(20.0)	0.011	4(40.0)	6(60.0)	1.000
Female	45(84.9)	8(15.1)	0.001	59(69.4)	26(30.6)	0.332	19(86.4)	3(13.6)	0.030	22(81.5)	5(18.5)	0.062
Age [year, (%)]												
18-44	24(72.7)	9(27.3)	0.001	23(60.5)	15(39.5)	0.069	8(100.0)	0(0)	0.002	12(63.2)	7(36.8)	0.142
45-59	17(68.0)	8(32.0)	0.021	36(51.4)	34(48.6)	0.309	8(66.7)	4(33.3)	0.150	7(63.6)	4(36.4)	0.243
≤60	18(94.7)	1(5.3)	0.013	38(90.5)	4(9.5)	<0.001	11(91.7)	1(8.3)	0.112	7(100.0)	0(0)	0.109
Operation time [min, (%)]												
≤12	13(72.2)	5(27.8)	0.012	22(47.8)	24(52.2)	0.557	1(50.0)	1(50.0)	1.000	11(73.3)	4(26.7)	0.018
>12	46(78.0)	13(22.0)	0.001	75(72.1)	29(27.9)	0.001	26(86.7)	4(13.3)	0.002	15(68.2)	7(31.8)	0.445

## Discussion

In this study, we found that people with the intestinal morphologies of tortuousness and dissociation seem to more often experience constipation, especially those with tortuousness, than the general population. Moreover, further analysis using subgroups did not attenuate this relationship.

The association of constipation with colonoscopy diagnostic results for diseases such as CRC has been an area of particular interest in the constipation field in recent years (17, 18). However, little is known about the relationship between constipation and other elements of the colonoscopy procedure, especially the characteristics of intestinal morphologies. This is, to our knowledge, the first study to present data from a Chinese hospital on the association of constipation and characteristics of intestinal morphologies. In this report, we evaluated this association in 510 patients and further explored it by using subgroups in order to provide a valuable reference of the characteristics of intestinal morphologies and their effect on constipation.

Multiple factors typically converge in the pathology of constipation, including intrinsic factors, behavioral factors, and environmental factors (19, 20). A review of the literature on studies conducted in North America suggests that the prevalence of constipation is consistently higher in women than in men and increases gradually with age, particularly after the age of 70 years (21). A possible contributing factor to the higher rates in females may be higher risk of injury to the pelvic floor muscles and nerves that are required for defecation (22) following childbirth (23). In addition, most epidemiologic studies demonstrated a higher prevalence of constipation in elderly individuals (1, 5, 24), due in part to intrinsic changes in colonic motility and physiology in the elderly, such as an increase in collagen deposition in the left colon (25), which predisposes individuals to developing constipation (24).

Moreover, gastrointestinal disorders such as enteritis may play a part in the onset of constipation (26). Parthasarathy et al. (2016) found that mucosal microbiota might affect epithelial and mucosal function, and achieved 94% accuracy for discriminating between healthy individuals and constipated individuals by examination of fecal and colonic mucosal microbiota (27). In addition, the quality of colon cleansing directly affects the ability of the clinician to perform a high-quality examination because of difficulty in visualizing mucosa, which affects the diagnostic yield (28). Thus, the BBPS and colonoscopy diagnosis were included in the adjusted model.

Furthermore, the prevalence of constipation varies between countries even when uniform criteria are used. Consequently, environmental, cultural, ethnic, dietary, or genetic factors may influence the incidence of constipation (29). In this study, almost all patients came from Zhongshan, Guangdong Province, China, which minimized the geographical variability. In our opinion, such conditions also minimized the effect of the association between geographical factors and constipation. Consequently, we did not incorporate geographical factors into our model. This might cause some interference with the results and is a shortcoming of the study.

Defecography is one of the most common examinations performed on constipated patients. This examination can show the shape of the anorectal and partial colon region, but generally fails to fully reflect the complete state of the colon. A colonoscopy serves as a common endoscopic examination and intervention procedure (30), and it can provide the possibility for novel observations. Abnormalities of intestinal morphologies may be causal agents that hinder or delay fecal emptying, thereby contributing to constipation, but the association of the characteristics of intestinal morphologies as determined by colonoscopy and their link to constipation is not well studied. To this end, we designed the study to answer the question: “Do the characteristics of intestinal morphologies as determined by colonoscopy affect constipation or not?” Some preliminary conclusions were drawn, but verification by other studies is still needed.

There were several limitations to this study. Owing to the limitations of its design, the other risk factors for constipation, such as sport activity and low fluid intake, were absent from the data collection. The selection of variables was underrepresented, resulting in limitations for the model. In addition, the sample size may have been insufficient, which could have deviation in the statistical analysis. A larger sample size and multicenter study is needed for further research and evaluation. In addition, the majority of the enrolled patients in the constipation group were female; although this is consistent with the epidemiology of constipation and normal in general population studies of constipation, it may have impacted the generalization of the characteristics of intestinal morphologies for men with constipation. Despite these limitations, we hope that our results can be informative for the selection of treatment methods for constipation.

## Conclusion

This study showed that compared with the general population, people with intestinal morphologies of dissociation and especially tortuousness seem to more often experience constipation. Our study provides accumulated data on constipation, and we believe that our results are a valuable resource for the study of constipation.

## Data availability statement

The raw data supporting the conclusions of this article will be made available by the authors, without undue reservation.

## Ethics statement

The studies involving humans were approved by the Institutional Review Board (the local Ethics Committee of the hospital) and the registration number for this study was 2019ZSZY-LLK-039. The studies were conducted in accordance with the local legislation and institutional requirements. The participants provided their written informed consent to participate in this study.

## Author contributions

YX: Writing—original draft. QX: Writing—review and editing.
